# Selective and complementary use of Optical Coherence Tomography and Fluorescein Angiography in retinal practice

**DOI:** 10.1186/s40662-016-0058-2

**Published:** 2016-10-17

**Authors:** Samir S. Shoughy, Igor Kozak

**Affiliations:** 1 The Eye Center and The Eye Foundation for Research in Ophthalmology, PO Box 55307, Riyadh, 11534 Saudi Arabia; 2 King Khaled Eye Specialist Hospital, Riyadh, Saudi Arabia

**Keywords:** Retina, Imaging, Optical coherence tomography, Fluorescein angiography

## Abstract

The current field of posterior pole and retinal imaging of the human eye has expanded into detailed analyses of the macula, retinal periphery, individual retinal layers, vitreoretinal interface, imaging of the choroid and the optic nerve head. The challenge in retinal imaging is the enduring pursuit of deeper penetration into tissues, increased resolution to the cellular level, and interpretation of observations. How much deeper can we go and with what resolution and reproducibility? These are fundamental questions for experts in search of novel imaging modalities. New discoveries may resolve existing controversies, but inevitably stimulate new questions. Emerging technologies in retinal imaging include adaptive optics retinal imaging and optical coherence tomography-based retinal angiography.

In this review, the focus of our discussion will be the discrepancy between the findings (interpretation) of one imaging technology that do not agree or are not even found with a complementary technology. If a clearly seen abnormality is present with one technology but absent in another, what are the possible explanations? Following is a summary of key concepts of retinal and optic nerve imaging modalities and current controversies regarding their interpretation and/or limitations.

## Background

The two most common imaging modalities utilized to detect macular edema due to various etiologies include fluorescein angiography (FA) and optical coherence tomography (OCT). Fluorescein angiography for clinical use was first described by Novotny and Alvis in 1961 [1]. The retinal vessels and leakage are detected by photographing the retina after injecting sodium fluorescein into the subject’s peripheral veins. Since that time, and with improvements, it has been routinely used in the clinical practice to analyze retinal (and choroidal) vascular diseases. Traditional camera-based systems use blue light to illuminate the retina and detect fluorescein emission. Enhancements to this have surfaced in recent years through the use of confocal scanning laser ophthalmoscopy (cSLO) that emits a very precise laser wavelength designed to achieve peak emission from the fluorescein. There is an improved signal-to-noise ratio and less out-of-focus and scattered light when using cSLO [2].

Since its introduction, optical coherence tomography has rapidly assumed an important and indispensable role as a noninvasive diagnostic and monitoring imaging modality in numerous diseases of the retina and the choroid [3]. Optical coherence produces cross-sectional images of optical reflectivity in the retina using optical interferometry to resolve the distances of reflective structures within tissue [4]. New generations of this technology, including spectral-domain and swept source OCT, have enabled even faster acquisition and reduction in the signal-to-noise ratio [5]. The introduction of OCT angiography and increasing experience with interpreting OCT images has reduced the need for FA and its associated intravenous injection.

Both FA and high-definition OCT imaging are used to illustrate different pathologic aspects. FA identifies the anatomical location and pattern of vascular leakage, and is generally a qualitative and functional study, whereas OCT allows a morphological and quantitative assessment of pathology such as macular edema (ME) by producing two or three-dimensional images of the retinal tissue. While both FA and OCT are used extensively to detect and characterize the same pathology, in some instances OCT and FA may yield discrepant findings in the same patient [6, 7]. In this review we will highlight some of these discrepancies and their underlying etiologies in different retinal and choroidal diseases.

## Review

### Controversies in detection of macular diseases

#### Age-related macular degeneration

Age-related macular degeneration (AMD) is the leading cause of severe visual impairment in elderly people in the developed world; however, the management of this condition has been radically altered by the introduction of anti-vascular endothelial growth factor therapy [8]. The visual prognosis in cases of neovascular AMD is variable and depends upon the location, composition, and size of the neovascular membrane. FA is still a mainstay in diagnosing neovascular AMD and providing information about the membrane size from the area of visible leakage.

Today, spectral domain OCT (SD-OCT) and non-invasive fundus autofluorescence imaging can provide additional anatomical information beyond the FA, including visualization of the layer of the retina and choroid involved with the neovascular membrane [9, 10]. OCT has gained popularity for the diagnosis and management of AMD because of its non-invasive nature and ability to produce cross-sectional images of the neurosensory retina, the subretinal space, retinal pigment epithelium, Bruch membrane and inner choroid. Mokwa et al. studied the agreement and activity of choroidal neovascularization (CNV) between spectral-domain OCT and FA in AMD [10]. They defined activity on FA if classic or occult CNV leakage was detected and on SDOCT if diffuse or cystoid intraretinal fluid or subretinal fluid accumulation was seen. The agreement between SD-OCT and FA regarding the activity of CNV lesions in their study was found in 79 %. They felt that when the two technologies did not agree, it was explained by the fact that FA and SD-OCT imaging provided different information about retinal pathology. The FA is used to obtain information about the perfusion and the growth of new vessels as well as the integrity of the blood-retinal barrier. OCT provides detailed information about anatomical and pathological changes in the layers of the retina, such as the presence of cystoid spaces. Cystoid spaces on SD-OCT may not necessarily indicate active CNV but may represent structural defects resulting from chronic disease. In these cases, the disagreement between SD-OCT and FA will be evident because fluorescein leakage on FA will not be noted [11].

On the other hand, CNV activity may be seen on FA and not be apparent on SD-OCT; this occurs if only intraretinal cystoid spaces and subretinal fluid accumulation are considered to represent CNV activity on SD-OCT. Other findings were found to indicate CNV activity, including intraretinal hyper-reflective flecks and fluid accumulation in the sub-RPE space, such as serous components of a pigment epithelial detachment (PED) [10].

Kozak et al. also reported a discrepancy in the detection of macular edema in various retinovascular diseases including AMD. They observed in 1,272 eyes that 94.97 % had the finding of ME or subretinal fluid confirmed by both techniques. There were 3.86 % of eyes in whom the FA showed dye leakage in the macular area and OCT demonstrated a normal foveal contour. Conversely, in 1.17 %, OCT showed intraretinal and subretinal fluid, which was not revealed by FA [6]. Most recently, Wilde et al. reported diagnosing 278 eyes with choroidal neovascularization (CNV) using SD-OCT and 231 diagnosed with FA [12]. In their cohort, they found a total of 47 false positive cases with SD-OCT (16.9 %) and reported sensitivity and specificity of SD-OCT alone for detecting CNV 100 % and 80.8 %, respectively. They concluded that SD-OCT was highly sensitive for detecting newly presenting neovascular AMD; however, in comparison to the reference standard of non-stereoscopic fundus FA in a specialized clinic, it did not seem accurate enough to replace FA in the diagnosis of wet AMD [12]. A report by Moult et al. using swept-source OCT angiography (OCTA) in exudative AMD demonstrates visualization of CNV in 16 of 19 eyes with previously confirmed exudative AMD [13].

The use of OCT has increased dramatically during the past decade, whereas use of FA had declined considerably [14]. Despite the high sensitivity of SD-OCT to identify CNV and CNV activity, many believe that FA still has a role in the initial diagnosis and staging of eyes with CNV. FA allows visualization of retinal vasculature and neovascular retinal/choroidal proliferations in addition to dynamic features such as perfusion and exudation [13, 14].

The American Academy of Ophthalmology AMD Preferred Practice Pattern advocates the use of intravenous fundus FA when a patient complains of new metamorphopsia or has unexplained blurred vision; it can also be used when the clinical examination reveals elevation of the RPE or the retina, subretinal blood, hard exudates, or subretinal fibrosis. Fluorescein angiography is also indicated in the following situations: to detect the presence of and determine the extent, type, size, and location of CNV; to detect persistent or recurrent CNV following treatment and to assist in determining the cause of visual loss that is not explained by the clinical examination [15, 16]. The use of FA and/or OCT is determined in part by the treatment strategy used for a particular patient. As stated in a recent editorial, both modalities are needed to comprehensively monitor patients with neovascular AMD [16]. The retina and imaging community is excited to see if improvements in OCT angiography, which currently lacks the component of dye leakage, will be the technology that will entirely eliminate intravenous fluorescein angiography.

## Uveitis

Macular edema is one of the most common vision threatening complications of uveitis and can affect patients with different types of ocular inflammation [17]. Cystoid macular edema (CME), the most common structural type of uveitic macular edema (UME), was noted in 33 % of all uveitis patients [18, 19]. If the media is clear, the presence of macular edema can be detected clinically. However, biomicroscopic evaluation of macular edema may be difficult when the amount of the fluid and the anatomical changes are minimal or there is an inflammatory reaction in the eye. Fundus FA is the conventional method for the assessment of UME by visualizing the area of leakage. It is particularly valuable to assess the retinal vascular integrity and to characterize the area of the foveal avascular zone. OCT imaging is non-invasive and directly assesses macular thickness and the presence of intraretinal cysts [17].

Several studies evaluated the agreement between FA and OCT when diagnosing macular edema in patients with uveitis. Kempen et al. found that OCT and FA offered only moderate agreement regarding macular edema status in uveitis cases. The discrepancy noted in some cases was attributed to the difference in what FA and OCT measure. Macular leakage detected by FA and macular thickness as measured by OCT are caused by the same pathology but have different impacts on our diagnosis and treatment [20]. Leakage may be noted on FA in the absence of detectable macular thickening on OCT, which may occur in either a severely damaged macula that is atrophic and thin but has ongoing inflammatory leakage, a macula where leakage has recently begun and thickening has not yet occurred, a macula distorted by epiretinal membrane but not thickened, or a macula where leakage is balanced by physiologic outflow of fluid from the macula providing a steady state without thickening. On the other hand, macular thickening may occur in the absence of leakage on FA. This may occur for reasons other than current vascular leakage like failure to clear osmotically active molecules from the retina or dysfunction of the retinal pigment epithelial pump, and might take some time to clear after leakage is no longer present [20]. They also found that in most cases of uveitis, OCT seemed the most appropriate first test to evaluate the macula, unless some other specific indication for FA exists (e.g., suspected vasculitis, vascular closure, neovascularization or optic nerve leakage, or for evaluation of a white dot syndrome). This was based on the observed superiority of OCT in obtaining gradable images, along with its lower cost, safety, better tolerance and non-invasive nature. They suggested that obtaining the second test after a negative result on the first seems justified when detection of macular edema would alter management [20]. Ossewaarde-van Norel et al. reported on the discrepancy between FA and OCT findings in 46 % of uveitic eyes [21]. They found FA leakage in the absence of macular edema on OCT predominantly in eyes with birdshot chorioretinopathy, which also exhibited lower central retinal thickness values compared with the other uveitis entities. On the other hand, they found macular edema on OCT in the absence of FA leakage in association with intermediate uveitis. Interestingly, they also found that the consistency between FA and OCT was noted frequently in patients with active uveitis; OCT positive findings in absence of FA leakage was common in eyes with inactive uveitis [21]. Similar to the study by Kozak et al. [3] the OCT technology used in the two reports was time-domain.

While OCT seemed like the most appropriate first test to evaluate the macula in patients with uveitis, FA was found more sensitive in detecting CNV secondary to inflammatory conditions such as multifocal choroiditis [18]. Another limitation of OCT imaging is the lack of identification of vasculitis or vascular occlusions in cases of uveitis. Kotsolis et al. demonstrated that FA was more reliable in documenting actively proliferating neovascularization than either time- or spectral-domain OCT in patients with uveitis [18]. They recommend that FA should be used in the evaluation of eyes suspected of having active CNV in MFC and in any other diseases exhibiting inflammatory-related neovascularization of the macula. They found clear evidence of actively proliferating neovascularization on OCT in only 53.8 % of the eyes that clearly demonstrated CNV by FA. They explained this discrepancy by accumulation of blood and proteinaceous exudate in the subretinal space in the inflammatory-related CNV. The inflammatory debris appear to produce a homogeneous cellular mass, which fills the sub-neurosensory space surrounding the CNV. Consequently, this highly reflective, serosanguineous material gives a false impression of attachment between the RPE and the retina with OCT imaging and the absence of the usual silent reflectance indicative of detachment. Other possible explanations for this discrepancy include the possibility that the OCT image did not register areas that were noted to be leaking during the FA; and leakage with FA may not always be associated with subretinal or intraretinal bulk flow of fluid [22, 23].

## Diabetic macular edema

Prior to the development and widespread use of OCT technology in ophthalmology, clinical examination with contact lens and FA were employed commonly in diabetic patients to evaluate, plan treatment, and to monitor diabetic macular edema (DME). Because clinical detection of macular edema may be possible only if severe macular involvement has already developed, it is then mandatory to image the macula in suspicious cases since early diagnosis of macular edema leads to more effective treatment. Both imaging modalities, FA and OCT, are complementary investigations, each revealing different aspects of the pathophysiologic aspects of DME [24]. Fundus FA demonstrates the area of dye leakage, while OCT quantifies macular edema. Spectral OCT has some advantages over FA in the evaluation of DME, including shorter image acquisition time, lack of systemic risks, and reproducible quantitative reporting of retinal thickness measurements. In patients with DME, minute structures including microexudates can be mapped and quantified [25].

In a comparison of FA and OCT for detection of ME in diabetic patients, Ozdek et al. found that the sensitivity of OCT was higher than that of FA, especially for the cystoid pattern of ME [26]. Accordingly, OCT can facilitate treatment decision and follow-up of diabetic patients, which is especially important in the early stages of diabetic maculopathy when the structural changes are not yet evident with slit-lamp biomicroscopy or FA. Soliman et al. found that early morphologic changes in diabetic ME may be seen better with FA than with OCT [27]. The combined data from both OCT and fluorescein angiography may provide a clearer understanding of the anatomic and physiologic characteristics of clinically significant diabetic macular edema [28]. These studies have used time-domain OCT technology. Macular thickness measurements in patients with diabetic macular edema differ significantly in magnitude between TD-OCT and SD-OCT systems [29]. This is primarily due to the different definition of outer retinal border, and whether RPE is or is not included in the measurement. At the same time, repeatability of retinal thickness measurements with spectral-domain OCT has been shown to be comparable to the time-domain OCT [30–32].

Some reports, however, favor SD-OCT over fundus FA because of sensitivity. Jittpoonkuson et al. reported that SD-OCT gave earlier macular edema diagnosis than FA in 3.53 % of eyes in their study and that residual macular edema at follow-up visits was missed by FA in 1.18 % of eyes [33]. In a study by Brar et al. [34] spectral-domain OCT technology has shown presence of cysts in petalloid type of FA leakage. Diffuse non-cystoid angiographic macular edema showed microcysts on SD-OCT, but was more commonly associated with thickening or distortion of the retinal layers without cyst formation. It is, thus, possible that scanning laser SD-OCT allows visualization of cystic structures not seen on fluorescein angiography.

## Myopic choroidal neovascularization

Pathologic myopia, a major cause of visual impairment and legal blindness, is associated with progressive elongation of the globe. It may be accompanied by degenerative changes in the sclera, choroid, Bruch's membrane, retinal pigment epithelium (RPE), and neurosensory retina. Choroidal neovascularization at the macula is the most common vision-threatening complication of pathologic myopia [35]. The diagnosis of new-onset myopic CNV may be difficult to establish purely by fundus examination for several reasons, including choroidal pallor, posterior pole staphyloma, pigmentary changes, lacquer cracks, and chorioretinal atrophic lesions [36]. Both FA and SD-OCT are important diagnostic tools for myopic CNV. Fundus FA measures area of dye leakage and OCT demonstrates the amount of macular edema or outer retina disturbance.

Leveziel et al. found no agreement regarding signs of active CNV between OCT and FA. Exudative features of myopic CNV were more obvious on FA (82 %) than on SD OCT (48.6 %). They suggested that fluorescein angiography should be performed when new-onset myopic CNV was suspected. One possible explanation of the little information that can be obtained from OCT in cases of myopic CNV is the less exudative behavior of the new vessels associated with myopic CNV compared with those associated with neovascular AMD [37]. Another important factor less frequently mentioned in studies is sampling error in OCT scanning in highly myopic eyes, especially those with posterior staphylomas. Sampling error with incorrect placement of B-scans can significantly reduce the sensitivity of CNV detection. This is less common with the aerial view on fluorescein angiograms.

Despite proclaimed superiority of FA in cases of myopic CNV, Milani et al. found that, on the basis of FA alone, active myopic CNV can be misdiagnosed and they suggested the use of SD-OCT in combination with FA [38]. In a recent study, Chhablani et al. found that repeatability and reproducibility for the diagnosis of myopic CNV was better with FA compared with SD-OCT; however, SD-OCT was a comparatively better tool for ruling out the presence of myopic CNV [39].

Gaucher et al. described the OCT finding of a characteristic bulge of the macular retina, RPE, and choroid within the concavity of the moderate posterior staphyloma in myopic eyes. In their study, FA showed atrophic changes in the macular retinal pigment epithelium (RPE) in all eyes, combined with focal points of leakage in some eyes. The dome-shaped macula often is associated with RPE atrophic changes and foveal retinal detachment, which may explain the visual impairment in those eyes [40].

## Pseudophakic cystoid macular edema

Pseudophakic cystoid macular edema (pseudophakic CME, also termed “Irvine-Gass syndrome”) is a swelling of the fovea due to fluid accumulation occurring a few weeks to months after cataract surgery. It is one of the most important causes of visual impairment after cataract surgery. The prevalence of pseudophakic CME varies from study to study depending on the method used in its detection. By using fluorescein angiography, a prevalence of up to 20 % has been reported. However, with inclusion of loss of vision as a requirement to establish the diagnosis, the prevalence of pseudophakic CME was found to be only 2 %. Its incidence has also decreased with less invasive cataract surgery [41].

Fundus FA in cases of pseudophakic CME can reveal capillary dilatation and leakage from small perifoveal capillaries in the early phase of the angiogram. In later phases, pooling in the outer plexiform layer results in the classic perifoveal ‘petaloid’ staining pattern in addition to staining of the optic nerve due to capillary leakage [42]. In cases of pseudophakic CME, OCT can reveal the cystic spaces in the outer nuclear layer of the central macula to enable measurement of the macular edema. FA has been considered the gold standard for the diagnosis of pseudophakic CME.

In cases of difficult venous access, oral fluorescein administration is an alternative for obtaining a retinal angiogram. Barteselli et al. found that oral FA had better sensitivity compared with spectral-domain OCT for detection of pseudophakic CME [7]. The general consensus, however, is that OCT is now the first method of choice because of its non-invasive nature and ability to evaluate and follow macular edema after cataract surgery. Compared to fundus FA, measurement of the macular thickness with OCT may provide better correlation with visual acuity than FA grading [41, 42]. Spectral-domain optical coherence tomography has been shown to detect retinal findings, including photoreceptor inner segment/outer segment junction (ellipsoid zone) abnormalities that are correlated with visual acuity changes in treated patients with Irvine-Gass syndrome [43].

## Central serous chorioretinopathy

Central serous chorioretinopathy (CSCR) is characterized by one or more serous detachments of the neurosensory retina, commonly associated with retinal pigment epithelium (RPE) detachments. The diagnosis of CSCR is mainly based on clinical diagnosis but may be aided by FA and OCT. Fluorescein angiography classically shows the leakage of dye from the choroid through a focal RPE defect and its pooling in the subretinal space in acute CSCR. Alternatively, OCT is used to quantify the amount and extent of the subretinal fluid, demonstrate thickening of the neural retina and is commonly used for monitoring during the follow-up and also for diagnosing the changes in the neurosensory retina [44–46].

Fluorescein angiography is essential for the identification of the leakage site for appropriate treatment with laser photocoagulation while OCT should be used for follow-up and evaluations of retinal thickness. On the other hand, SD-OCT can be crucial in detecting small amounts of fluid in chronic CSCR where the signal from depigmented RPE can make it difficult to discern such fluid. It is also useful in detecting CNV associated with CSCR [47].

OCT-based angiography is an emerging in vivo imaging modality that images retinal vessels and their filling without the use of intravenous dye injection. It captures physiological and pathological blood vessels at different layers of the retina by separating static/tissue from motion/blood flow signals [48]. The detection of CNV in CSCR using OCT-based angiography was recently reported in a pilot study. Choroidal neovascularization was diagnosed in 8 of 27 eyes (30 %) with CSCR based on FA imaging analysis. Optical coherence tomography angiography and corresponding OCT B-scans detected 100 % (8 of 8) of these CNV lesions and correctly excluded 100 % (19 of 19) of eyes with CSCR without CNV. Both sensitivity and specificity reached 100 % [48]. Additionally, a recent study by Maftouhi et al. has shown that OCT angiography allowed for the detection of CNV in chronic CSCR that is not visible with other imaging techniques such as indocyanine green angiography [49].

### Controversies in retinal nerve fiber layer, optic nerve and ganglion cell imaging

Imaging for glaucoma has been a topic of intense research, and has gained a prime position in the management of patients with glaucomatous optic neuropathy. Disc photographs have been considered the gold standard for documenting the optic nerve appearance. The limitations of photographs are the lack of quantitative data to demonstrate progression and the inability to provide early detection of glaucoma. Photography of the retinal nerve fiber layer (RNFL) has been proposed as a tool for detecting glaucoma. However, it was fraught with issues in developing the images to demonstrate the RNFL and has not evolved much in the digital age. Regardless, the ability to see the RNFL is important in assessing the amount of axonal loss due to ganglion cell death [50, 51]. Initial OCT images had limitations in seeing the RNFL, so scanning laser polarimetry was more popular for a little while [52, 53]. Simultaneous with the development of the scanning laser polarimetry (SLP) and OCT was the use of confocal scanning laser ophthalmoscopy (SLO) [54].

As the SLO, OCT and polarimetry were each used to image the RNFL, the initial winner was the nerve fiber analysis with polarimetry. The algorithms and normative database enhanced the usability of the instrument [55]. Polarimetry is sensitive to corneal birefringence and the average polarization did not always match a patient with the scans [56]. OCT seems to be less affected by these properties, so it most likely provides more accurate RNFL thickness data and is not prone to spurious birefringence readings. The issues in obtaining useful RNFL scans then turned to the normal anatomy and the orientation of the RNFL radiating out of the disc. For instance, a myopic patient may have a significant tilt, with a RNFL pattern that differs from a hyperope. This tilt moves the peaks a few clock hours temporally, resulting in an “out of synch peak” in place of the classic RNFL peak at the 6 and 12 position [57]. Thus, the RNFL normative data does not accommodate the variation induced by tilt. This can really only be picked up by three dimensional imaging or MRI [58]. A small crowed disc would have a large bundle exiting the disc. Many of the “artifacts” in RNFL measurements are most likely due to the lack of adjustments for normal RNFL anatomical variation. Another factor is the size of the circle used to determine the peaks of the RNFL (the TSNIT plot). If it is small and close to the rim, we are less likely to see the effects of RNFL bundle loss due to bundle loss. The problem with the larger circle is the relationship to possible disc tilt. If there is a large tilt and the scan is away from the nerve, it may have a larger “out of synch” peak [59].

Optic nerve photos provide images of the cup-to-disc in single plane image or stereo-images. Even amongst experts the cup appearance did not always match the level of visual field damage. Methods for detecting focal rim changes have included the “ISNT” rule [60]. Studies validating this rule showed moderate sensitivity but low specificity [61]. Nerve imaging devices do not seem to be much better in detecting damage than photographs. The Heidelberg Retinal Tomogram (HRT) has various modes for analyzing the rim and determining the likelihood of glaucoma. Another method is the Moorfields Regression Analysis (MRA). The MRA and the ISNT rule have been evaluated in their ability to detect glaucoma. There seems to be some evidence that the sensitivity and specificity using the HRT are good at detecting glaucoma [62]. The OCT was also evaluated for its ability using the ISNT rule and was not found to have the sensitivity or specificity of either photos or HRT from prior studies [63, 64]. This is likely related to how OCT defines the cup. The way the cup is measured and defined has been evolving with OCT and the methods for determining the neural retinal rim (NRR) are improving. Initially, the cup-to-disc from the Stratus OCT did correlate well with photos, direct evaluation of the disc or HRT. OCT was the outlier [65]. The reference for the disc has been changed and incorporates the tilt of the disc and variants in the slope of the disc into the overall cup-to-disc assessment. The end of Bruch’s membrane has been set as a reference below the disc margin in most machines. The real refinements are how the cup is determined beyond that. The internal limiting membrane and the shortest arc length or distance from the end of the RPE become the point set for the cup [66, 67]. This has greatly improved the ability to see the cup in a similar ratio to what is seen clinically. The cup-to-disc, however, is still not 100 % specific or sensitive, so attention is still paid to both the RNFL and ganglion cells to determine the damage from glaucoma.

For many years, many have agreed that ganglion cell loss seems to be the source of optic cupping and RNFL loss due to glaucoma. Because the macula gives the impression of having an abundance of ganglion cells, this was the location of initial efforts at looking for glaucoma-induced cell loss. The first generations of OCT did not have the resolution to discriminate the ganglion cell layer very well [68]. With the SD-OCTs there has been much progress in looking at the ganglion cell layer in glaucoma. It is now equivalent to imaging the RNFL and ONH in evaluating glaucoma [69]. Asymmetry has been shown to be a sensitive indicator for early glaucoma [70]. Because the central portion of the visual field is affected from the macular ganglion cell loss, structural and function correlation needs to shift toward using the more central part of the visual field [71].

Diabetic retinopathy has been considered primarily a retinal microvascular disorder caused by the direct effects of hyperglycemia and by the metabolic pathways it activates [72]. However, some recent studies have demonstrated that thinning of the inner retina may develop in patients with diabetes, even before visible vascular signs of diabetic retinopathy [72–74]. The SD-OCT may represent an indispensable tool for identifying early signs of neurodegeneration in diabetic patients, which may require neuroprotective measures [75]. Such findings cannot be obtained using fluorescein angiography.

## Emerging technologies

Adaptive optics is a relatively new tool that makes it possible for the ophthalmologist to evaluate the retina at a cellular level. Adaptive optics technology aims to correct the ocular aberrations and enhance performance of the optical system [76]. In addition to the axial resolution provided by the spectral-domain optical coherence tomography, adaptive optics provides diffraction-limited lateral resolution by correction of ocular aberrations [77]. The lateral resolution enables visualization of the photoreceptors, blood vessels, RPE, lamina cribrosa, RNFL and details of the optic nerve head [77, 78]. However, normative databases need to be developed for different populations as a reference for disease states [76]. Furthermore, the reproducibility of the obtained images will need to be confirmed before the full application of this technology in the clinic [79].

Optical coherence tomography angiography is a new, non-invasive imaging system that generates volumetric data of retinal and choroidal layers. It has the ability to show both structural and blood flow information. It allows the visualization of retinal microvasculature by detecting intravascular blood flow, using a split-spectrum amplitude-decorrelation angiography (SSADA) algorithm, without any dye injection [80]. OCT angiography has been investigated in various ocular diseases, including diabetic retinopathy, age related macular degeneration, retinal vascular occlusions, polypoidal choroidal vasculopathy, macular degeneration, macular telangiectasia type 2, choroideremia, sickle cell disease, central serous chorioretinopathy, CNV in myopia and peripapillary retinal perfusion in glaucoma [80–85]. OCT angiography has several compelling characteristics including its noninvasive nature. It can be acquired in a few seconds, compared to several minutes for FA. The 3D imaging allows for depth resolution of pathology and separation of individual vascular layers for evaluation [86]. Its current limitations include a relatively small field of view, inability to show leakage, and tendency for image artifacts [80].

## Conclusion

In conclusion, FA and OCT are complementary investigations. OCT provides detailed imaging of anatomical retinal layers, allows detection of microstructural changes, and helps perform quantitative assessment during follow up. FA is used to evaluate retinal vascular perfusion and the integrity of the inner blood-retinal barrier. Therefore, the use of either or both imaging modalities is advisable for the most accurate diagnosis and the proper management of retinal diseases.
